# Mechanochemical Preparation of Biomass-Derived Porous Carbons

**DOI:** 10.3390/molecules30153125

**Published:** 2025-07-25

**Authors:** Jerzy Choma, Barbara Szczęśniak, Mietek Jaroniec

**Affiliations:** 1 Institute of Chemistry, Military University of Technology, 00-908 Warsaw, Poland; jerzy.choma@wat.edu.pl; 2 Department of Chemistry and Biochemistry & Advanced Materials and Liquid Crystal Institute, Kent State University, Kent, OH 44242, USA; jaroniec@kent.edu

**Keywords:** biomass, mechanochemistry, porous carbons, biowaste-derived carbons

## Abstract

Conventional methods for the synthesis of porous carbons are typically time- and energy-consuming and often contribute to the excessive accumulation of waste solvents. An alternative approach is to employ environmentally friendly procedures, such as mechanochemical synthesis, which holds great potential for large-scale production of advanced carbon-based materials in coming years. This review covers mechanochemical syntheses of highly porous carbons, with a particular focus on new adsorbents and catalysts that can be obtained from biomass. Mechanochemically assisted methods are well suited for producing highly porous carbons (e.g., ordered mesoporous carbons, hierarchical porous carbons, porous carbon fibers, and carbon–metal composites) from tannins, lignin, cellulose, coconut shells, nutshells, bamboo waste, dried flowers, and many other low-cost biomass wastes. Most mechanochemically prepared porous carbons are proposed for applications related to adsorption, catalysis, and energy storage. This review aims to offer researchers insights into the potential utilization of biowastes, facilitating the development of cost-effective strategies for the production of porous carbons that meet industrial demands.

## 1. Introduction

**Figure 1:**
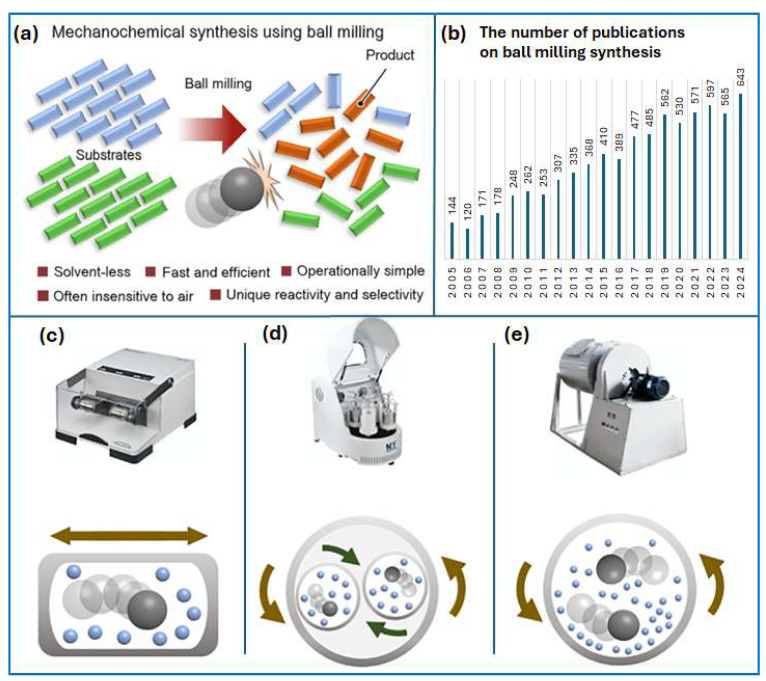
(**a**) Schematic illustration of mechanochemical reaction via ball milling. (**b**) Number of publications on ball milling synthesis in recent years (data from Web of Science^™^). A set of milling devices, including schematics of the motion of the milling media. These vary from (**c**) the small-scale mixer mill (<10 g scale) to (**d**) the medium-scale planetary mill (<100 g scale) and (**e**) the production-scale rolling mill (kg scale). Adapted with permission from Ref. [1]. Copyright © 2020, Elsevier Inc.

Mechanical energy can be used to induce changes in the structure and physicochemical properties of condensed matter (typically solids), leading to chemical transformations (Figure 1a) [1]. According to the International Union of Pure and Applied Chemistry (IUPAC), a mechanochemical reaction is one that is triggered by absorption of mechanical energy [2]. Recently, mechanochemistry has re-emerged as an interesting field of chemical synthesis because it enables efficient and fast synthesis of inorganic and organic materials under solvent-free or low-solvent conditions [3]. Therefore, mechanochemistry can be considered as an environmentally friendly method. Moreover, using mechanochemistry may result in new reaction pathways and/or mechanisms that differ from conventional chemical processes [4]. Mechanochemistry may complement conventional activation methods, namely through heat, radiation, or electrochemistry [5]. Since 2005, the number of publications on ball milling synthesis has significantly increased (Figure 1b). Simultaneously, a large variety of commercially available milling devices has emerged (Figure 1c–e).

Mechanochemistry is closely related to tribochemistry, where chemical reactions take place at the interfaces between different environments. Although interest in mechanochemistry has recently grown, it still seems to be much less studied and understood compared to conventional methods of energy delivery. We believe that mechanochemistry may become a more widely adopted technique in the future for two main reasons. First, there is growing evidence that mechanochemistry can be both effective and advantageous across various areas of synthesis and manufacturing. Second, our current reliance on solvents is unsustainable, as it contributes to the generation of liquid waste, much of which is derived from fossil resources. For instance, solvents account for about 85% of the chemicals used in the pharmaceutical industry. Even with typical recovery rates of 50–80%, the environmental impact remains significant [4]. Consequently, the use of various solvents can pose significant hazards and be energy-consuming in terms of production, purification, and recycling. In mechanochemistry, solvents are either not used or are used in negligible amounts.

At present, the majority of chemicals, fuels, and materials utilized by humans are derived from non-renewable fossil resources such as petroleum, natural gas, and coal. While the global demand for energy, chemicals, and materials continues to rise, accessible fossil fuel reserves are steadily diminishing. The reliance on fossil resources not only contributes to severe environmental pollution and global climate change but also introduces economic instability due to fluctuations in petroleum-based fuel prices, which hinder sustainable development. Consequently, there is an urgent need to identify renewable alternatives for the production of chemicals, fuels, and materials [6].

Porous carbons have emerged as highly valuable materials in various energy and environmental applications, including catalysis, electrochemical energy storage, and gas adsorption and separation [7,8]. In recent years, nanoporous carbons have become highly attractive materials for electrodes in supercapacitors or hybrid devices [9]. High-performance energy storage has become possible thanks to novel nanocarbons such as graphene, carbon onions or carbon nanotubes, etc. [10]. Although these materials show promising performance in future devices, their synthesis is often energy- and time-consuming and currently not feasible at ton production levels. In the industry, activated carbons are currently the most widely used porous materials. They possess high specific surface areas due to the presence of micro- and mesopores; however, they often fall short in delivering the superior energy storage performance exhibited by more advanced nanoporous carbons, such as graphene-based materials or ordered mesoporous/macroporous carbons [11,12].

Biomass is the most abundant renewable resource on the planet, with an estimated global production of approximately 130 billion tons per year [13]. Through biochemical and/or thermochemical processes, biomass can be transformed into sustainable chemicals, fuels, and materials [14]. According to the International Energy Agency, biomass could contribute up to 10% of global energy needs by 2035, and by 2050, it may supply as much as 27% of transportation fuels [15]. Biomass-based energy is considered environmentally friendly due to its carbon neutrality and minimal ecological impact. Furthermore, waste biomass can be converted into biochar, a sustainable and ecological material suitable for applications such as chemical synthesis, wastewater treatment, and energy-related technologies [16]. The use of mechanochemical methods to produce functional porous carbons from bio-organic precursors seems to be a sustainable and attractive approach. Therefore, researchers seeking new materials and optimal synthesis conditions have explored mechanochemical techniques for the preparation of valuable porous carbons from biomass and have proposed their applications in adsorption, separation, catalysis, electrocatalysis, and electrochemistry, among others [6,11]. Compared to conventional solution-based methods (e.g., sol–gel, self-assembly, hydrothermal synthesis), the mechanochemical method has the advantage of solvent-less and operationally simple processing, involving fewer synthesis steps. Therefore, mechanochemical approaches, such as ball milling, offer a promising and scalable route for the production of porous carbons. This review highlights the synthesis of porous carbonaceous materials via ball milling from biomass-based (waste) precursors and their versatile applications. It briefly discusses general aspects of mechanochemical synthesis of biomass-derived carbons, their physicochemical properties and applications in adsorption, catalysis, electrochemical, and energy-related applications. Finally, the opportunities and challenges of the mechanochemical synthesis of porous carbons from biomass are also discussed.

## 2. Mechanochemical Synthesis

In mechanochemistry, researchers explore chemical transformations driven by mechanical actions such as compression, shearing, or friction. In these processes, the energy needed to initiate reactions is provided through mechanical force, analogous to how heat, light, and electrical potential serve as energy sources in thermochemistry, photochemistry, and electrochemistry, respectively. Mechanochemistry has a long-standing history and remains highly relevant due to its ability to efficiently and quantitatively promote solid-state reactions, often without the use of solvents or with only minimal solvent involvement, as seen in wet milling and liquid-assisted grinding. In more conventional chemical synthesis, solvents play a crucial role in dispersing energy, dissolving/solvating and transporting chemicals. Therefore, using insufficient amounts of solvents may result in hindered mass and energy transport. Despite advances in reducing solvent usage in mechanochemical synthesis, they are still usually needed in the post-synthesis process of isolating products in a satisfactorily pure form.

### 2.1. History of Mechanochemistry

Apparently, the history of mechanochemistry can be traced for nearly 25 centuries [17]. According to Takacs et al. [18], the earliest documented mechanochemical reaction was the grinding of cinnabar (HgS). Theophrastus of Eresus (371–286 B.C.), wrote in his book “De Lapidibus” (“On Stones”) that “Native cinnabar was rubbed with vinegar in a copper mortar with a copper pestle yielding the liquid metal.” This represents a prominent example of a mechanochemical process. It is unknown why the mechanochemical synthesis of mercury from sulfide was forgotten in the Middle Ages. However, the medieval literature also contains other mechanochemical processes, described between 300 B.C. and the 18th century. Agricola et al. [19] documented several examples of chemical reactions induced by mechanochemical action related to mining and metallurgy. Interestingly, in the 17th century, Bacon and Rawley mentioned four treatments that remain among the most important procedures for preparing active solid substances, one of which was grinding [20]. From an interesting history of mechanochemistry [17], a few facts are worth mentioning. Faraday described the reduction of silver chloride by grinding it in a mortar with zinc, tin, iron, or copper in 1820. He referred to this process as the “dry way” of inducing chemical reactions. According to his observations, the reaction between silver chloride and zinc was rapid and highly exothermic, suggesting a self-sustaining mechanochemically induced process. However, the first documented experiment explicitly combining mechanical action with a chemical reaction is attributed to Lea in 1866. In his research, mercury and silver chlorides decomposed during mortar grinding, despite typically melting or subliming without decomposition when heated. This was a foundational moment in the formal study of mechanochemical reactions.

In modern mechanochemistry, the launch of the “International Conference on Mechanochemistry and Mechanical Alloying (INCOME)” marked a significant milestone. The first INCOME conference took place in Košice in 1993. The conference was organized by the International Mechanochemistry Association (IMA), an associate member of IUPAC; the IMA was founded in 1988 in Tatranská Lomnica, Slovakia.

### 2.2. Fundamentals of Mechanochemistry

The term mechanochemistry is often broadly defined and includes any chemical reaction initiated by mechanical energy, such as grinding, and it is also used in this review in this sense. However, Kaupp [21] argues that this broad usage is inaccurate. He suggests that the term should be reserved for cases where mechanical energy directly breaks strong chemical bonds, such as in polymers or single molecules, leading to the formation of reactive centers (often radicals) that undergo further reactions. Under this narrower definition, grinding-induced reactions—primarily driven by increased surface contact and mixing—would not qualify as true mechanochemical processes. IUPAC defines a mechanochemical reaction (with a hyphen) as “a chemical reaction induced by direct absorption of mechanical energy,” [21]. Although the note provides guidelines for its use in the context of polymers, the fundamental definition is broader and does not include any restrictions related to the atomic-scale mechanisms involved. Therefore, the common usage of the term appears justified. It is also worth noting that most authors write the term mechanochemical without a hyphen.

Grinding usually refers to a mechanical action that reduces particle size using manual (e.g., mortar and pestle) or automated methods, like ball milling or extrusion. While often associated with solvent-free conditions, mechanochemistry can involve small amounts of liquid. Even a minimal solvent amount can significantly enhance or enable reactions, a process now commonly termed “liquid-assisted grinding” (LAG). LAG improves reaction efficiency compared to dry milling and is considered a suitable modern method equivalent to “kneading” [17].

There may be some misunderstanding about the expression “solvent-free.” This indicates that no solvent was intentionally introduced into the reaction, highlighting the practical advantage of this approach. However, solvents can be introduced with solid starting reactants, e.g., using hydrated metal salts, molecular solvates, or moisture. Therefore, although the use of the term “solvent-free” is often accurate in a practical sense, caution is needed, especially in proposing mechanochemical reaction mechanisms. The solvent-free reaction in the general context does not necessarily correspond to a completely solvent-free process.

### 2.3. The Importance of Controlling the Mechanochemical Process

It is essential to understand and control the parameters of mechanochemical reactions [5]. Three key parameters influence mechanochemical reactions: the kinetic energy of the balls before collision, the way this energy is transferred to the reactants, and the collision frequency. The kinetic energy determines the maximum energy available for transfer during ball milling. These distinct modes of energy transfer can lead to different reaction outcomes [22].

The mechanochemical process can be controlled by adjusting the parameters related to the device and accessories used, as well as their various settings. For instance, the most significant parameters affecting the efficiency of the ball milling process are milling speed and time, reaction atmosphere, the mass ratio of balls to the milled sample, and the type of device (mill). The degree of vessel-filling influences the trajectories of the balls and thus the transfer and delivery of energy to the milled substances. The effect of the degree of fill of the milling vessel with balls was studied in a Knoevenagel condensation reaction of vanillin with barbituric acid [23]. In this experiment, it was observed that the optimal filling of the milling vessel with balls was about 25% of the total vessel volume. However, it is important to emphasize that this may not necessarily apply to other reactions or other types of mills. Milling accessories (balls and vessels) are made from various materials, including Teflon, aluminum oxide, zirconium oxide, stainless steel, and tungsten carbide.

Mass transfer is rarely a problem on a small scale, and it can be more easily controlled in solution by stirring compared to milling. Parameters that are commonly varied during the optimization of reaction conditions, such as stoichiometry, reaction time, and temperature, are critically important in mechanochemical processes. Among these, temperature is particularly challenging to control during ball milling, especially in typical automatic devices. The reaction vessel heats up because of collisions of milling balls, and the temperature rise depends on the degree of fill of the vessel with milling balls and reactants, the type of sample, the size of the vessel, and the rotation speed (oscillation frequency). As an exception, in the case of cryogenic mills, liquid nitrogen automatically cools the vessel, but these are not commonly used in chemical processes [24].

Recent advances in ball mill design have focused on maximizing energy transfer, with high-energy mills now reaching speeds up to 2000 rpm and featuring improved vessel designs [25]. These instruments are usually equipped with water cooling systems, where the energy released during milling is dissipated through cooling, preventing the sample from overheating. Milling speed directly affects the kinetic energy of the balls—higher speeds result in greater energy input into the reaction system.

### 2.4. Scalability of a Mechanochemical Process

Any synthesis processes, in order to be useful in industry, such as those involving the decomposition or creation of molecules, must be scalable. In mechanochemistry, each type of milling device should allow the process to be carried out at different scales. For instance, in typical mills, gram-scale processes can be performed, which are suitable for laboratory research. For larger quantities of products, larger planetary mills, for example, are available in various sizes and configurations, whereas very large, stirred mills are available for pilot and production scales. For instance, the Metso HIGmill™ stirred mill has a capacity of 50,000 L and can process materials weighing over 1000 kg [25]. Stolle et al. [26] presented a scalable mechanochemical Knoevenagel condensation reaction between vanillin and barbituric acid using a planetary mill, increasing the scale from 20 to 300 mmol. This enabled the production of about 80 g of product in a short time with good reaction efficiency. Another approach to scaling up involves converting a batch process into a continuous one, e.g., by using extruders instead of mills. Extruders push material through confined spaces using shear and compressive forces. For example, James et al. [27] synthesized MOFs at a rate of several kilograms per hour using twin-screw extruders. Thus, it can be confidently stated that significant progress has been made in developing specialized equipment for mechanochemical processes. Readers are encouraged to see the recent reviews in [28,29,30], which compare various mechanochemical synthesis methods in the context of biomass processing, including aspects such as efficiency, energy consumption, scalability, and limitations.

## 3. Biomass

Biomass is a readily available, renewable, and relatively inexpensive organic substrate, making it an extremely important and inexhaustible resource for designing and manufacturing sustainable functional materials. Recent studies on biomass-derived carbons have shown that our strong dependence on non-renewable carbon precursors can be significantly mitigated by utilizing biomass [31,32]. Biomass is the second-oldest energy source after the sun.

**Figure 2:**
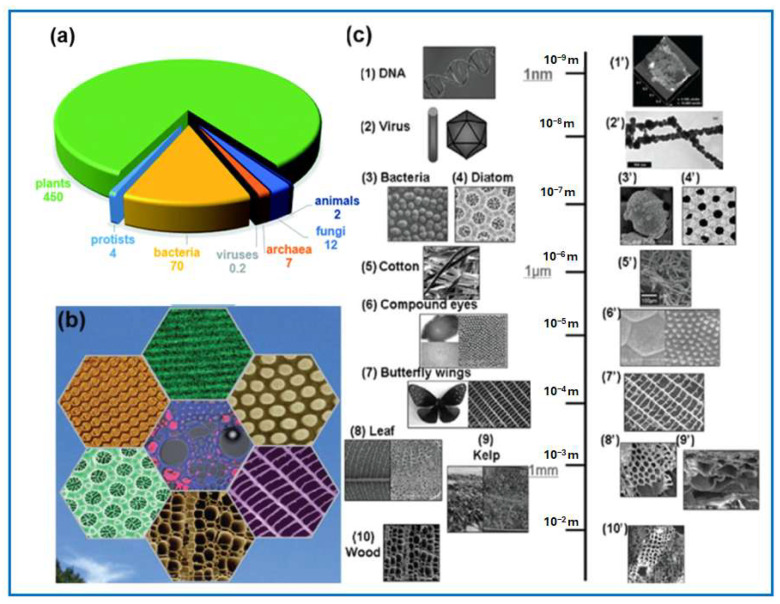
(**a**) Graphical illustration of the global biomass distribution. Values in the graph are expressed in gigatons of carbon of biomass [11]. Reproduced with permission from Ref. [11]. Copyright © 2020, The Royal Society of Chemistry. (**b**) Various microstructures from biomaterials in nature. (**c**) Overview of biological templates, placed on a length scale according to their critical dimensions. On the left-hand side are the original biological structures, whereas on the right-hand side are the corresponding synthesized templated structures [33]. (**b**,**c**) Reproduced from Ref. [33], and licensed under CC-BY.

Global biomass totals about 550 gigatons of carbon, with 80% from plants and 15% from bacteria; other organisms make up less than 10% (Figure 2) [11,33]. Around 60% of biomass is on the Earth’s surface [34]. Biomass is a major carbon reservoir, including forest and agricultural wastes, industrial and household biowastes, and marine waste, offering diverse carbon sources with varied composition and structure [35]. However, direct carbonization of biomass, such as wood, agricultural waste, and animal waste, usually leads to carbons with a low specific surface area and heterogeneous pores in terms of size and shape [36]. The improvement towards porosity development and pore accessibility of biomass-derived carbons is usually achieved through an additional activation process.

### 3.1. Main Types of Biomass

**Figure 3:**
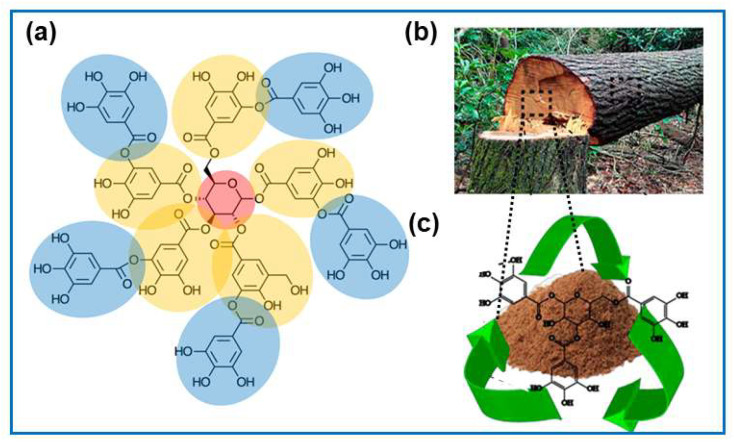
Molecular structure of (**a**) tannic acid—glucose center in red, intermediate esterified gallic acid unit in yellow, and esterified gallic acid end group in blue [40]. Reproduced with permission from Ref. [40]. Copyright © 2021, American Chemical Society. (**b**) Tannins are widely distributed in the bark of trees. (**c**) Tannin powder is prepared by vacuum drying after purification, following their initial extraction from bark using an aqueous solvent [38]. Reproduced with permission from Ref. [38]. Copyright © 2017, American Chemical Society.

This section covers common biopolymers, their extraction, and their applications. Tannins—redox-active polyphenols found widely in plants—are easily extracted from wood waste at room temperature and biodegrade naturally [37,38,39,40]. They are classified into hydrolyzable tannins (gallotannins, ellagitannins, complex tannins) and condensed tannins (proanthocyanidins), with (+)-catechin and tannic acid as key examples (Figure 3).

**Figure 4:**
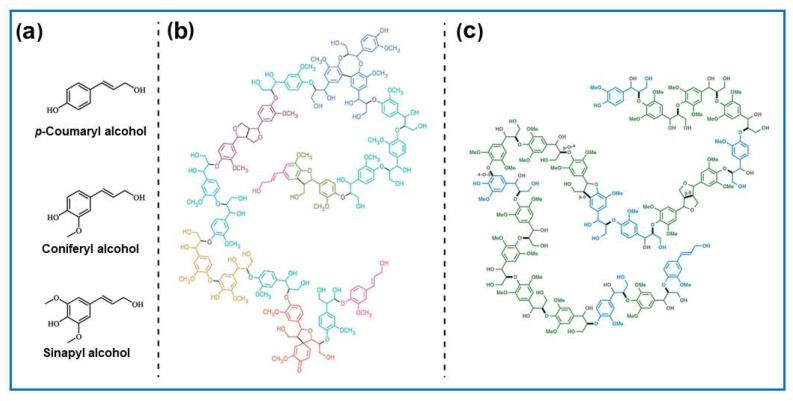
(**a**) Structures of monolignols, the primary building blocks of lignin; (**b**) a softwood lignin model; (**c**) a hardwood lignin model [41]. Reproduced with permission from Ref. [41]. Copyright © 2020, Wiley-VCH Verlag GmbH & Co. KGaA, Weinheim.

Lignin acts as a binder in plant cell walls, constituting about 10–25% by weight of lignocellulosic biomass [41]. It is a complex mixture of cross-linked amorphous copolymers derived from p-coumaryl, coniferyl, and sinapyl alcohols, which differ in their methoxy group content (Figure 4). Recently, lignin has attracted significant attention as potential biomass for various applications, e.g., to obtain advanced carbon-based materials. Moreover, it is one of the renewable biopolymers characterized by a high degree of aromaticity and thus can be regarded as a good source of value-added aromatic chemicals.

Lignin’s monomer units are linked primarily by β-O-4 glycosidic bonds and contain various functional groups, including methyl ethers, phenols, carbonyl groups, carboxyl groups, and hydroxyl groups. Consequently, it serves as an interesting alternative for obtaining a wide range of low-molecular-weight products and materials based on their arenes. The significant complexity of lignin’s structure also poses a major challenge to its processing. Since the interest in lignin’s structure and utilization is vast, we encourage readers to see other reviews that cover more detail on this topic [42,43]. The value of lignin as a biorefinery raw material depends on how easily it can be extracted and its chemical structure, which allows for further processing [44]. Many studies cover isolating lignin from lignocellulosic biomass and depolymerizing lignin to produce biofuels and chemicals [44,45]. Due to the intrinsic chemical structure of lignin, the obtained carbons show good performance in energy storage, catalysis, and pollution removal.

The composition of biomass varies significantly. Lignocellulose typically constitutes the largest portion of the biomass and is composed of cellulose (C_6_H_12_O_6_)_n_ (38–50%), hemicellulose (C_5_H_8_O_4_)_n_ (20–35%), lignin [C_9_H_10_O_3_(OCH_3_)]_n_ (15–25%), and mineralized inorganic substances [45]. Lignocellulose is a raw material to produce biochemicals, bioethanol, and biofuels.

Cellulose, the most abundant natural biopolymer, is produced by plants at a rate of about 75 billion tons annually [44]. It forms structural microfibrils in plant cell walls, composed of linear β-(1,4)-D-glucose chains with crystalline and amorphous regions linked by hydrogen bonds. Nanocellulose, derived from cellulose via chemical or mechanical processes, has recently attracted significant interest [46]. Nanocellulose exhibits distinctive properties such as high tensile strength (10 GPa), high stiffness (up to 220 GPa modulus of elasticity), a high Young’s modulus (~150 GPa), low density (1.6 g/cm^3^), modifiable surface chemistry, transparency, flexibility, and a low thermal expansion coefficient [47]. These attractive characteristics make NC an ideal material for different applications, such as in biomedical devices, solar panels, filtration membranes, food packaging, paints, and coatings [48,49]. As one of the main polymers in the biosphere, cellulose is a non-toxic, renewable, and cost-effective source for producing sustainable carbon materials. Depending on its origin, cellulosic biomass exhibits slight variations in its chemical composition, leading to different reactions during pyrolysis, which result in the formation of the carbon skeleton of biochar.

Hemicellulose is a heteropolysaccharide that constitutes about 20–35% of lignocellulose and surrounds cellulose in plant cell walls. It forms covalent, hydrogen, and ionic bonds with other components. Composed of short, branched chains of various sugars (pentoses, hexoses, hexuronic acids, and deoxyhexoses), it binds to cellulose microfibrils via hydrogen bonds and van der Waals forces [50]. By cross-linking with lignin, hemicellulose provides structural support to the cell wall and acts as a “glue” within individual fibers. Compared to cellulose, hemicellulose has lower thermal and chemical stability because of its lack of crystallinity and lower degree of polymerization. As a result, hemicellulose can be easily hydrolyzed, yielding glucose, xylose, galactose, mannose, arabinose, glucuronic acid, and mannuronic acid. Unlike cellulose, the structure of hemicellulose is highly variable and lacks uniform repetition. Properties of hemicellulose depend on its chemical composition [28]. The chemical hydrolysis and decomposition of hemicellulose have been extensively studied for various applications, including the production of ethanol as a renewable fuel additive, as valuable chemicals for food additives, and applications in the pharmaceutical and mining industries [51,52,53].

Starch is a plant-based polysaccharide that serves as a key energy reserve and is abundant in staple crops like corn, potatoes, rice, tapioca, and legumes (e.g., peas, lentils, and fava beans). Starch extracted directly from plants is known as “native starch”, while “modified starch” refers to those altered through biological, chemical, or physical processes to enhance specific properties. Structurally, starch granules (2–100 μm) consist of concentric growth rings (120–500 nm), made up of blocklets (20–50 nm) formed from amorphous and crystalline lamellae (9 nm). These contain amylose and amylopectin chains (0.1–1 nm). Overall, starch is insoluble in cold water and has a density of 1.5 g/cm^3^. Its general formula is (C_6_H_10_O_5_)_n_, composed of glucose monomers, which can be organized to form two distinct macromolecules: amylose and amylopectin [54].

### 3.2. Biomass Pretreatment

Lignocellulosic biomass is the most abundant biomass on Earth. Its conversion into chemicals and fuels is very difficult because of its complex structure. Pretreatment is therefore essential to break down and remove lignin from biomass, disrupt cellulose crystallinity, and improve access to cellulose and hemicellulose for hydrolysis. Many studies have shown various pretreatment methods for different biomass types [55,56].

**Figure 5:**
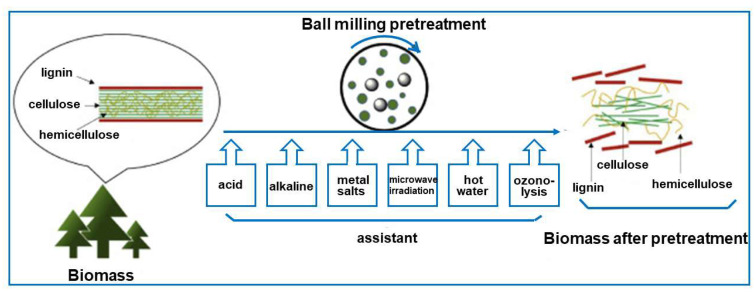
Biomass pretreatment solely with mechanical milling or in the presence of assistants [6]. Reproduced with permission from Ref. [6]. Copyright © 2020 Elsevier Ltd.

Diluted acids and alkalis are the most used solvents for the pretreatment of lignocellulosic biomass, but their use generates large volumes of waste. Currently, ball milling is considered as an efficient and environmentally friendly method for the pretreatment of lignocellulosic biomass [6]. Ball milling-based pretreatment can significantly reduce biomass particle size by altering its crystalline structure, decreasing the degree of cellulose polymerization, and loosening chemical bonds. Ball milling can be used as a standalone pretreatment method or in combination with other techniques (such as microwave treatment or ozonolysis), as illustrated in Figure 5.

Mechanical milling is widely used in the pretreatment processes of biomass to convert it into valuable products [56,57,58,59]. Ball milling as a physical method eliminates the use of harmful chemical reagents. It has been observed that ball milling pretreatment significantly impacts biomass conversion. For instance, Zakaria et al. [58] utilized oil palm biomass to produce glucose and xylose through enzymatic hydrolysis. In this study, after dry ball milling of an empty fruit bunch (EFB) precursor in a planetary ball mill for 60 min at 250 rpm, the biomass was directly hydrolyzed with *Acremonium cellulase*. The maximum glucose and xylose yields reached 67.5% and 80.1%, respectively, which were much higher than those from the untreated EFB (15.9% and 5.4%, respectively). Zhang et al. [60] reported that ball milling pretreatment of pre-fermented rice straw led to their highest ethanol yield compared to other methods, including microwave-assisted and ultrasound-assisted alkali pretreatments of the biomass. Jiang et al. [61] compared the effects of pretreatment with ionic liquid and ball milling on glucose yield from cellulose during enzymatic hydrolysis. The glucose yield from untreated cellulose was only 20.9%, whereas from ball-milled cellulose, it was about 84.5%, and from ionic liquid-treated cellulose, it was 78.0%. Cellulose subjected to either ball milling or ionic liquid pretreatment exhibited a much higher susceptibility to enzymatic hydrolysis. Moreover, it was highlighted that ball milling pretreatment is more environmentally friendly and operationally straightforward. Pre-milled samples can be further processed without the need for washing and filtration steps. For example, conventional pretreatment methods of rice straw using sodium hydroxide and aqueous ammonia resulted in material losses of 34.2% and 14.8%, respectively. In contrast, no biomass loss was observed in the case of ball milling pretreatment [59]. Furthermore, ball milling pretreatment does not generate any by-products, whereas chemical pretreatments produce phenolic and heterocyclic compounds that may inhibit subsequent processes (e.g., fermentation) [62]. The high efficiency of lignocellulosic biomass conversion via ball milling results from the breakdown of its rigid structure by mechanical forces like impact, compression, shearing, and friction [63]. This enhances enzyme or catalyst accessibility to cellulose and hemicellulose. Ball milling induces key structural changes, including reductions in crystallinity index, polymerization degree, particle size, and thermal stability while increasing porosity [57,64,65]. Planetary ball milling has been shown to significantly alter the morphology and structure of cellulose, as confirmed by wide-angle X-ray diffraction analysis [64]. The material of the milling balls and bowls plays a crucial role in ball milling efficiency. Studies show that using alumina, zirconia, or steel balls effectively reduces particle size and crystallinity of corn stover during pretreatment [65]. Steel balls were the most effective at pulverizing the biomass. Therefore, milling balls with a high density are considered more suitable for biomass pretreatment prior to enzymatic hydrolysis. Recently, a comprehensive review covering mechanochemical processing of starch was reported [66]. Ball milling was shown as an effective method to reduce the starch particles to a nano size, enhance water percolation into its molecules (swelling), incorporate oxygen functional groups, and/or convert hydroxyl groups into carbonyl and carboxyl groups (oxidative effect). Ball milling can also be used to modify starch properties such as viscosity, gelatinization temperature, retrogradation, stability, solubility, etc.

Compared to other lignocellulosic biomass pretreatment methods, ball milling offers key advantages, including its simple operation, reduced solvent use, and minimal post-treatment requirements. Performing reactions under solvent-free conditions may enhance reaction rates and lower energy consumption. Additionally, the mechanical energy generated during ball milling can trigger chemical reactions, making mechanochemical depolymerization a promising starting step for biorefinery applications. This strategy is particularly advantageous for solid catalysts, as it helps overcome the mass transfer barrier between solid–solid interfaces. For instance, weakly acidic solid catalysts (functionalized with -COOH or -OH groups) can effectively convert waste biomass into chemicals through ball milling, thus eliminating the need for the use of corrosive concentrated H_2_SO_4_. Parameters such as milling time, rotation speed, size and material of the balls, and milling mode significantly influence the efficiency of this process, including selectivity of product formation [66].

## 4. Conversion of Biomass Waste into Porous Carbons

The porosity and, hence, functionality of porous carbons are usually assessed through adsorption–desorption isotherms of nitrogen at −196 °C or argon at –186 °C. These isotherms, preceded by vacuum degassing at an elevated temperature, allow for the calculation of the specific surface area (SSA) using the Brunauer–Emmett–Teller (BET) theory within the relative pressure range of 0.05 < *p*/*p*_0_ < 0.2. The value of the SSA can also be determined from adsorption isotherms using the density functional theory (DFT) method [67]. The total pore volume is calculated from the adsorbed gas volume at *p*/*p*_0_ = 0.99, assuming that micro- and mesopores are filled with liquid adsorbate [68]. According to IUPAC, porous materials are categorized as follows: (i) microporous materials (pores of 0–2 nm), (ii) mesoporous materials (pores of 2–50 nm), and (iii) macroporous materials (pores > 50 nm). Nanoporous materials, a subset of porous materials, typically exhibit high porosity, with pore sizes in the range of 1–100 nm.

Numerous organic materials classified as biomass have been used over several decades to produce a variety of carbon materials. Waste products containing biomass-derived matter can be generally classified as waste biomass, which serves as a readily available source for porous carbon materials [68,69,70,71,72,73,74,75,76,77,78,79]. For instance, activated carbons were prepared from almond shells through a two-step thermal treatment, carbonization, and carbon dioxide activation [69]. The activation process led to the development of micropores and a substantial increase in the SSA in the resulting carbon material. Two biologically similar by-products from the food industry, namely plum and peach stones, were used as precursors for the preparation of activated carbons [70]. The carbons produced via carbonization followed by CO_2_ activation were compared with those produced through a single-step CO_2_ activation process. The porosity of the obtained activated carbons was analyzed based on the following adsorption measurements: N_2_ (at −196 °C), CO_2_ (at 0 °C), i-butane (at 0 °C), and para-nitrophenol and methylene blue (both in aqueous solution at 25 °C). Peach stone-derived activated carbons exhibited a molecular sieve effect for i-butane, indicating the presence of narrow micropores in their structure. In contrast, activated carbons derived from plum stones showed significantly more developed microporosity and macroporosity, particularly when single-step CO_2_ activation was applied. Elsewhere, activated carbons were prepared from carbonized olive stones using either carbon dioxide or steam as the activating agent [71]. The pore structure of the resulting carbons was analyzed using nitrogen adsorption, carbon dioxide adsorption, and mercury porosimetry. The results indicated that carbon dioxide facilitated the opening and subsequent widening of narrow micropores. In contrast, steam activation led to carbons with expanded micropores and a lower overall micropore volume. In another study, five carbon samples were prepared from olive pomace biomass using KOH activation [72]. The microstructure of the carbon materials was tailored by varying the KOH-to-precursor weight ratio from 1:1 to 5:1. Detailed structural and morphological characterization (N_2_ and CO_2_ adsorption, X-ray diffraction, and scanning electron microscopy) revealed that as the proportion of the activator increased, the resulting carbons transitioned from predominantly microporous to micro–mesoporous structures. The increased SSA in the activated carbons resulted in higher hydrogen adsorption at high hydrogen pressures, reaching hydrogen storage capacities under 200 bar up to 6 wt.% and 1.22 wt.% at −196 °C and 25 °C, respectively. These findings suggest that carbons derived from olive pomace may be promising materials for hydrogen storage. Highly porous granular activated carbons were prepared from the dried endocarp of coconut shells via both CO_2_ physical activation and chemical activation with H_3_PO_4_ or ZnCl_2_ [73]. The results indicate that the physical activation process allowed a more precise control of pore size distribution, while chemical activation resulted in a lower mass loss, higher mechanical strength, and higher volume of mesopores in the resulting carbon materials. Microporous carbons can also be prepared from coconut shells via a one-step CO_2_ carbonization and activation process [74]. Applying different time lengths of CO_2_ activation, the SSA and micropore volume of the obtained carbons varied in the ranges of 686–1327 m^2^/g and 0.31–0.65 cm^3^/g, respectively. These carbons reached CO_2_ adsorption capacities of 3.9 and 5.6 mmol/g at 760 mmHg and at 25 °C and 0 °C, respectively. These values of CO_2_ adsorption are typical for plant-derived carbons. In [75], the potential use of cork as a biosorbent and sustainable precursor for activated carbon was explored. Activation was carried out by impregnation with phosphoric acid followed by pyrolysis in a nitrogen atmosphere. The resulting materials were used for the removal of sunflower oil emulsified in water. Activated carbons with higher SSAs and fewer acidic surface groups exhibited an enhanced capacity for sunflower oil adsorption. Other microporous carbons were obtained from African palm shells [76]. Their preparation involved carbonization in a nitrogen atmosphere followed by activation with KOH. The micropore volumes (and SSAs) of the resulting carbons ranged from 0.16 cm^3^/g (365 m^2^/g) to 0.82 cm^3^/g (1890 m^2^/g), depending on the KOH-to-carbon ratio used in the activation process. These carbons exhibited high CO_2_ adsorption capacities at 1 bar. The study described in [77] aimed to produce activated carbons from empty fruit bunches (EFBs) from Malaysia and investigate their hydrogen storage capacity. The precursor was activated using physical or chemical processes. The SSAs and microporosity of the produced activated carbons ranged from 305 to 687 m^2^/g and up to 94%, respectively. Hydrogen storage capacity was examined at –196 °C and under pressures ranging from ambient pressure to 100 bar. Among the carbons studied, the EFB activated with 2 M KOH showed a maximum hydrogen adsorption capacity of 2.14 wt.% at 20 bar. Low-cost activated carbons with a high microporosity were obtained from walnut shells via a simple, one-step carbonization process combined with chemical activation using KOH [78]. Carbon activated at 800 °C in a nitrogen atmosphere exhibited a highly developed porosity (SSA of 1868 m^2^/g) and a large micropore volume of 0.94 cm^3^/g. Its CO_2_ adsorption capacity at 1 bar was as high as 9.54, 5.17, and 4.33 mmol/g at 0 °C, 25 °C, and 40 °C, respectively. The results showed that their CO_2_ adsorption capacity mainly depended on their internal microporous structure. In another work [79], it was shown that a direct carbonization of dried bread at 900 °C for 3 h in an inert atmosphere led to porous carbon with an SSA of about 1020 m^2^/g. Furthermore, the carbon could be activated using 1 M KOH solution at 600 °C for 1 h, yielding activated carbon with a higher SSA of 1370 m^2^/g.

Carbons with high porosity and a fibrous microstructure may be attractive for some applications. However, achieving a controlled porous microstructure in carbon fibers is challenging and often requires complex and time-consuming synthesis methods. In [80], the synthesis of nanoporous carbon fibers with high porosity relied on the activation of spider silk (a natural biomaterial) with potassium hydroxide. The resulting carbon fiber had a high SSA of 2730 m^2^/g and a large pore volume of 1.56 cm^3^/g, respectively. It showed exceptional CO_2_ adsorption capacities, capturing 23.6 mmol/g and 15.4 mmol/g of CO_2_ at 0 °C and 25 °C under 25 bar, respectively. Additionally, the adsorbent exhibited a high CH_4_ adsorption capacity of 8.6 mmol/g at 0 °C under 25 bar and H_2_ adsorption of 4.1 wt.% at −196 °C under 25 bar. The material also demonstrated an excellent regeneration ability and stability. These results highlight the potential of spider silk-derived porous carbons for energy and environmental applications. The cited examples clearly demonstrate that various biomass wastes can be successfully utilized to produce a range of porous carbons with exceptional properties, including high porosity and superior adsorption capacities.

## 5. Mechanochemical Synthesis of Biomass-Derived Carbons

Biomass is considered a promising and sustainable precursor for the preparation of advanced carbon materials due to its high carbon content of about 45–50 wt.% [81]. Carbon materials can be obtained from biomass via various methods, including pyrolysis, hydrothermal carbonization, ionothermal carbonization, and ball milling-assisted synthesis [81,82]. Mechanochemistry can also be used to enhance the functionality of carbon-containing materials or improve production efficiency [63]. During the high-energy grinding or milling process, mechanochemical energy reduces particle size, increasing the total surface area of the sample. Additionally, the defects created may become reactive sites, determining specific surface properties of the milled product.

### 5.1. Tannin-Derived Porous Carbons

Plant-derived polyphenols, including hydrolysable tannins (e.g., gallic, digallic, and ellagic acid) and condensed tannins (e.g., mimosa tannin) are highly suitable for the preparation of ordered mesoporous carbons (OMCs) [83]. A typical mechanochemical synthesis of OMCs involves milling a soft template, such as the triblock copolymer F127, with tannins in a ball mill to form a mesostructured polymeric composite [84]. Metal salts, e.g., Zn(OAc)_2_, Ni(OAc)_2_, Mg(OAc)_2_, and Co(OAc)_2_, can be added as agents facilitating crosslinking and/or generating metal-containing OMCs. OMC is obtained after removing the soft template during subsequent carbonization in an inert atmosphere. In this process, metal nanoparticles can be reduced in situ and trapped in its pores. The resulting metal-doped OMCs are suitable for use as catalysts, adsorbents, or supercapacitor electrodes. Tannins are rich in polyphenolic compounds, which undergo reactions analogous to those of phenol or resorcinol during the copolymer-assisted self-assembly process [85]. The beneficial difference in comparison to these organic compounds is that the synthesis of tannin-derived OMCs can be performed in the absence of aldehyde under acidic or neutral conditions (e.g., 1 mol/L HCl) [86,87,88]. For instance, OMCs were successfully prepared using a soft templating method at the pH of natural tannins (pH = 4.2, without pH adjustment using an acid catalyst) [87]. It was demonstrated that a pH of 4.2 or lower during self-assembly had no significant effect on the structure and properties of the resulting OMCs. Solid-state synthesis of OMCs via the mechanochemical method is beneficial because of the reduced time needed for self-assembly and polymerization. Moreover, highly ordered mesoporous carbons can be obtained by ball milling of tannins and Pluronic-type copolymers with or without crosslinking agents [87,88,89].

Dai et al. [84] presented a simple mechanochemical synthesis of OMCs and Ni-decorated OMCs. The obtained carbons featured homogeneous and tunable mesopores ranging from ~4 to 10 nm. In this approach, the mesopore sizes could be adjusted by varying the synthesis conditions. The OMCs were characterized by large pore volumes (up to 0.96 cm^3^/g) and high SSAs up to about 1000 m^2^/g. The synthesis involved mechanochemical mixing of tannins with a PEO–PPO–PEO triblock copolymer (Pluronic F88, F87, F68, F38, P123, P103, P85, or P65) or nonionic surfactants (PEO-based Triton X-100 and Brij-78), along with the nickel precursor (salt). Table 1 presents the pore structure parameters of the OMCs and Ni-OMCs obtained with different triblock copolymers and carbonization temperatures, as determined from N_2_ adsorption isotherms. Small nickel nanoparticles of about 5.4 nm were embedded within cylindrical mesopores and exhibited high thermal stability even at 600 °C. Furthermore, Ni-OMC demonstrated high activity in the hydrogenation of large molecules [84].

**Figure 6:**
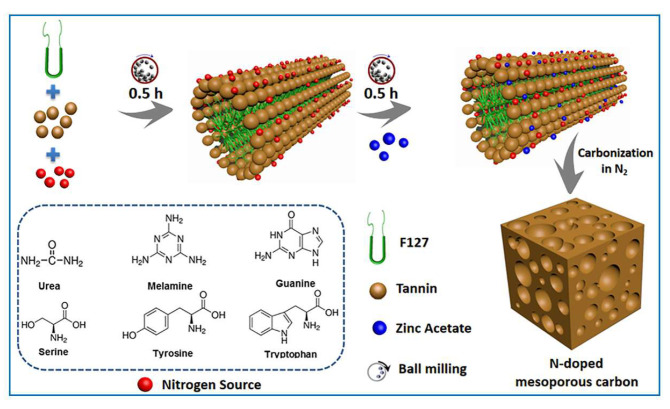
The schematic illustration of typical synthesis process of N-doped mesoporous carbon via one-pot mechanochemical synthesis using tannin as a carbon source [92]. Reproduced with permission from Ref. [92]. Copyright © 2019, Elsevier B.V.

More recently, Castro-Gutiérrez et al. [89] prepared highly ordered mesoporous carbons through the mechanochemical self-assembly of tannins using the triblock copolymer Pluronic F127 (PEO_99_–PPO_65_–PEO_99_) in the presence of a small amount of water, followed by carbonization at 900 °C for 3 h. By selecting an optimal ratio of components, i.e., tannin–Pluronic–water in a 2:1:2 ratio, a highly ordered mesoporous carbon with an SSA of 510 m^2^/g and mesopore volume of 0.24 cm^3^/g was obtained. Its two-dimensional hexagonal pore structure remained intact after thermal treatment at 1500 °C in an inert atmosphere. The authors demonstrated that the SSA of OMC could be successfully increased to ~1900 m^2^/g after its activation with CO_2_ for 75 min at 900 °C. The CO_2_ adsorption capacity of this carbon increased to 5.6 mmol/g after activation with potassium acetate at 800 °C for 1 h in a nitrogen flow. In [90], a fast milling-based strategy was implemented for the synthesis of Ru-OMC. The key chemical aspects of this synthesis were the use of mechanical force, which induced the coordination polymerization of a tannin–metal (Zn^2+^ and Ru^3+^), and hydrogen bonding interactions between the block copolymer (F127) and tannin. In the synthesis, Zn^2+^ ions diluted Ru^3+^ ions and prevented the sintering of Ru species, resulting in the formation of Ru nanoparticles no larger than 1.4–1.7 nm during carbonization (800 °C). The Ru-OMC catalyst showed good activity in the selective oxidation of benzyl alcohol to benzaldehyde using molecular oxygen. Elsewhere, in [91], the synthesis of metal oxide-doped mesoporous carbons involved ball milling of tannins and in situ thermal stabilization of the formed polymer with alkali metal oxides (e.g., MgO, CaO). The resulting porous carbons achieved surface areas of up to 520 m^2^/g and pore volumes of 0.65 cm^3^/g. In the work by Dai et al. [92], a solid-state synthesis of nitrogen-doped mesoporous carbons (N-MC) was proposed via mechanochemical mixing of tannins, a soft template, and an additional nitrogen source, e.g., amino acids, urea, melamine, or guanine (Figure 6). The nitrogen sources formed strong hydrogen bonds with the tannins, enabling effective nitrogen incorporation into the carbon framework during high-energy ball milling. The solvent-free synthesis makes this method a sustainable route for producing nitrogen-doped mesoporous carbon adsorbents. In this study, CO_2_ and light hydrocarbon adsorption performance of N-doped MCs was assessed via static adsorption experiments.

**Figure 7:**
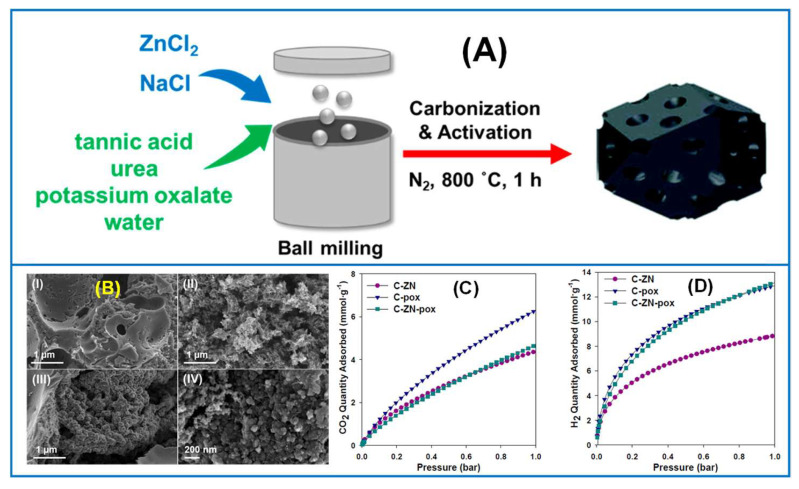
(**A**) Schematic illustration of the synthesis of tannic acid-derived activated carbons. (**B**) SEM images of (I) C-pox, (II) C-ZN-pox, and (III, IV) C-ZN. The as-prepared carbons were denoted as C-ZN-pox, where ZN refers to the eutectic salt and pox represents the activator used—potassium oxalate, K_2_C_2_O_4_. Additionally, C-pox was obtained using the same recipe as C-ZN-pox, except for the addition of ZN. (**C**) CO_2_ adsorption isotherms measured for the selected carbons at 0 °C; (**D**) H_2_ adsorption isotherms measured for the selected carbons at −196 °C [94]. Reproduced with permission from Ref. [94] and licensed under CC-BY.

In another study, tannic acid and urea, both low-cost and eco-friendly precursors, were used to synthesize nitrogen-doped porous carbon nanospheres via ball milling [93]. By adjusting the urea-to-tannic acid ratio and using a salt-templating method (NaCl/ZnCl_2_), researchers achieved tunable pore structures and a high nitrogen content (up to 8.83%) in the resulting carbons. Elsewhere, the mechanochemical salt-templating method was extended for the synthesis of highly porous activated carbons by adding mild chemical activators to the milling system [94]. The chemical activation with potassium oxalate and ZnCl_2_ produced highly porous carbons with surface areas up to 3060 m^2^/g and pore volumes of 3.07 cm^3^/g. These materials showed excellent gas adsorption capacities, making them promising for applications in adsorption and catalysis. For instance, the best sample exhibited high H_2_ (13.2 mmol/g at −196 °C) and CO_2_ (4.7 mmol/g at 0 °C) adsorption capacities under 1 bar (Figure 7).

**Figure 8:**
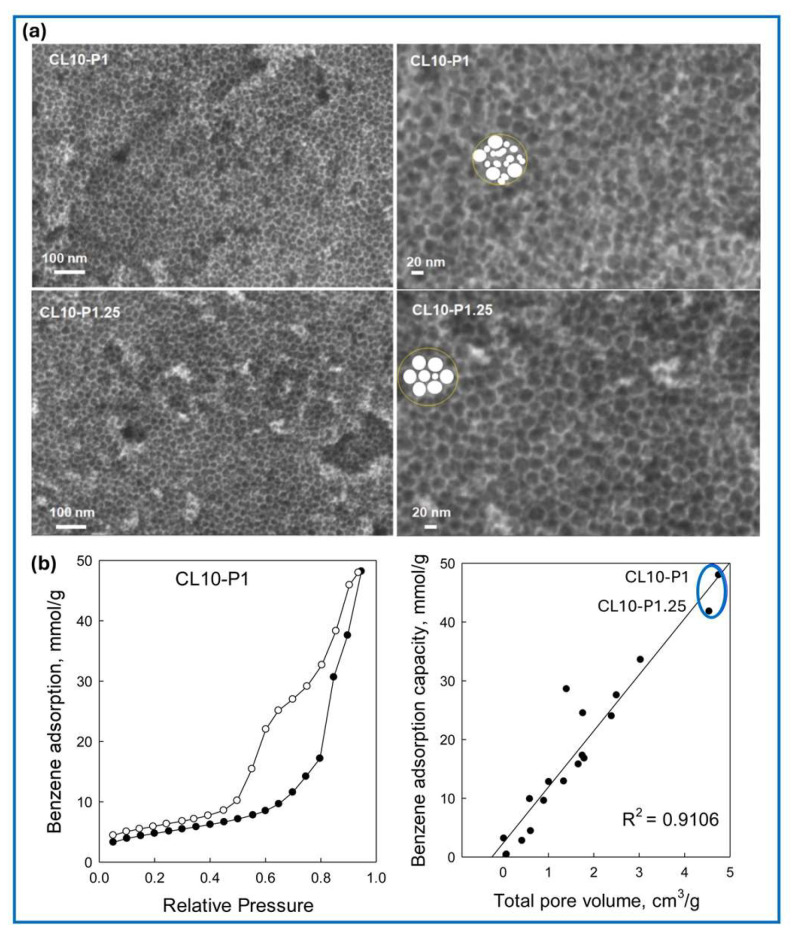
(**a**) SEM images of tannin-derived highly mesoporous carbons synthesized using ball milling-involved hard and soft templating methods (denoted as CL10-P1 and CL10-P.125, prepared with different amounts of soft template). (**b**) Benzene adsorption on CL10-P1 carbon with bimodal mesoporosity at 20 °C and the relationship between the adsorption capacity of different sorbents and their total pore volume; adsorption data from Refs. [96,97,98,99,100,101,102,103,104,105,106,107,108]. Reproduced with permission from Ref. [96]. Copyright 2024, Elsevier B.V.

Elsewhere, a coordination compound of tannic acid–Fe was synthesized by a simple mechanochemical method [95]. This compound was used as a precursor for the preparation of porous carbon for CO_2_ capture. Szczęśniak et al. [96] reported a facile mechanochemical synthesis of mesoporous carbons with uniform bimodal mesoporosity via a combined hard and soft templating strategy. Carbons synthesized by ball milling of tannins with Pluronic F127 and silica colloids, followed by thermal treatment at 750 °C and silica etching, achieved a high surface area (1218 m^2^/g) and bimodal mesoporosity with pore sizes of 13.8 and 24.8 nm (Figure 8a). The resulting material exhibited a large total pore volume of 4.75 cm^3^/g and a record-high benzene adsorption capacity of 48.0 mmol/g at 20 °C, corresponding to 375 wt% of benzene adsorbed on the carbon (Figure 8b). It was also shown that the total pore volume is a determining factor for benzene adsorption on different porous sorbents [97,98,99,100,101,102,103,104,105,106,107,108]. Ball milling can also be used in the facile and sustainable synthesis of graphitized mesoporous carbons [109]. This was achieved by the addition of iron(III) chloride to the milling system of a tannin and triblock copolymer Pluronic F127. This salt additive acted as a graphitizing catalyst during the subsequent direct carbonization process. In this work, it was shown that using a simple synthesis method, one can obtain carbons with a high degree of graphitization (up to 75%) and simultaneously have high SSAs of 360 m^2^/g and volumes of mesopores of about 0.40 cm^3^/g at a relatively low carbonization temperature of 750 °C. Interestingly, the resulting carbon was in the form of short nanotubes and spherical carbon nanocapsules. It was also demonstrated that the synthesis procedure of mesoporous graphitized carbons based on ball milling can be further used to obtain micro–mesoporous carbons with SSAs reaching 1100 m^2^/g by addition of potassium oxalate to the milling mixture in a ratio to tannin of 1:2 [109]. The resulting micro–mesoporous activated carbons effectively adsorbed about 4 mmol/g CO_2_ at 25 °C and 1 bar. Apparently, the iron salt plays a dual role in the synthesis of activated carbons, namely as a catalyst for graphitization and an additional micropore-generating agent (activator). It was experimentally determined that about 30% of the volume of ultramicropores in the obtained carbon originated from the activation with FeCl_3_, which under appropriate conditions acts similarly to the well-known activator ZnCl_2_. This study confirms a close relationship between effective CO_2_ adsorption and the volume of ultramicropores in the structure of the carbon adsorbent. This mechanochemical synthesis can be used to produce carbons with tunable porosity and suitable physicochemical properties.

### 5.2. Lignocellulosic Biomass-Derived Carbons

Lignin and cellulose are the most abundant renewable biomass sources, making them important renewable sources of carbon [110]. Moreover, lignin is a waste product from the paper industry. As mentioned above, lignin is a highly cross-linked, heterogeneous natural polymer with limited industrial use due to the lack of cost-effective methods for its depolymerization. This restricts its potential as a main source of valuable chemicals and carbon-based materials. However, there are many reports on the use of lignin to obtain activated carbons, lignin–silica composites, and related materials [111,112,113]. Direct carbonization of lignin followed by KOH or CO_2_ activation typically yields microporous carbons with little to moderate mesoporosity [114,115]. It has been frequently reported that post-synthesis activation is an effective method for developing microporosity in lignin-based carbons [111,115].

Nitrogen-doped nanoporous carbons derived from lignin were synthesized via solvent-free, mechanically induced synthesis [116]. This simple approach involved mechanochemical treatment and carbonization of a mixture of three solid materials: lignin, urea, and potassium carbonate. The resulting nitrogen-doped porous carbons exhibited very high SSAs up to 3000 m^2^/g and large pore volumes up to 2 cm^3^/g. The nitrogen-doped carbons were well suited for electrochemical energy storage as supercapacitor electrodes, exhibiting a high specific capacitance. This mechanochemical route seems to be scalable, time-efficient, and effective in the synthesis of highly porous carbons from lignin. The development of innovative methods for converting lignin into sustainable chemicals and functional materials is a key path to high-value utilization of lignocellulosic biomass. Carbon materials derived from lignin are highly promising for applications in energy and chemical engineering, catalysis, and environmental remediation. However, there are few reports on mechanochemical-assisted methods for converting lignin into carbon materials. For instance, the review in [113] summarizes the latest advancements in the controlled synthesis of lignin-derived carbon materials, including the mechanochemical approach. This study covers future research directions aimed at enhancing the usability of lignin-derived carbons for commercial applications. We believe that mechanochemistry may play a key role in the future processing of lignin.

**Figure 9:**
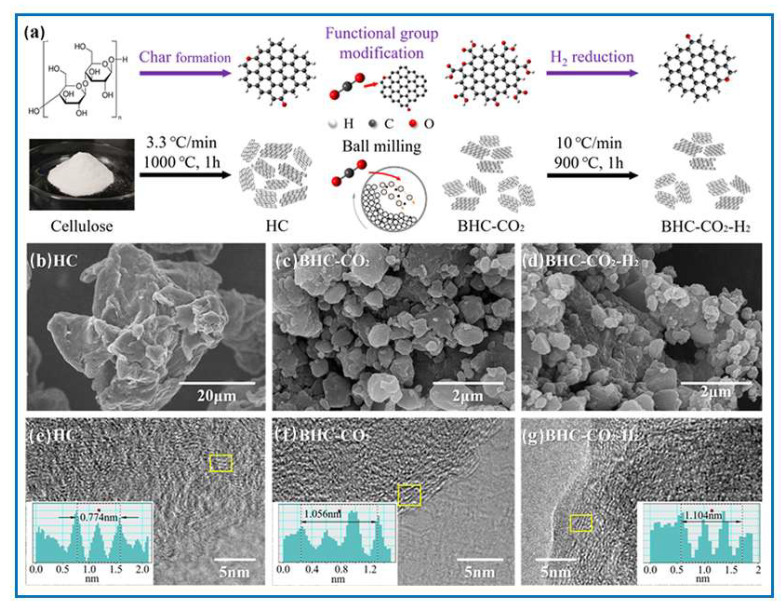
Preparation and microstructure of hard carbon (HC), the carboxyl group-modified hard carbon BHC-CO_2_, and the oxygen-scarce hard carbon (BHC-CO_2_-H_2_). (**a**) Schematic illustration of the preparation process from HC to BHC-CO_2_ and BHC-CO_2_-H_2_. SEM images of (**b**) HC, (**c**) BHC-CO_2_, and (**d**) BHC-CO_2_-H_2_. TEM images of (**e**) HC, (**f**) BHC-CO_2_, and (**g**) BHC-CO_2_-H_2_, including insets of Fast Fourier Transform (FFT) patterns [118]. Reproduced with permission from Ref. [118]. Copyright © 2019, American Chemical Society.

Cellulose is the primary component of biomass, and it is well suited for the cost-effective production of carbon materials. Over the past decade, cellulose-based carbons have been widely proposed for their use in energy storage systems [117,118,119]. The most commonly employed form of cellulose has been nanocellulose, including cellulose nanocrystals, nanofibrils, and bacterial nanocellulose [120,121]. In contrast, microcrystalline cellulose (MCC), a readily available commercial product, has been less extensively studied. However, in recent years, MCC has gained recognition as a promising precursor, particularly for the preparation of carbon electrodes in supercapacitors, owing to its cost-effectiveness and sustainability [117,118,122]. In [118], a mechanochemical process for producing modified hard carbon (BHC-CO_2_) from cellulose in the presence of dry ice was reported. The resulting material demonstrated a high oxygen content of 19.33 at.% with dominant carboxyl groups (Figure 9). This study demonstrated that oxygen-containing groups in carbon play a key role in sodium-ion storage. The BHC-CO_2_-based anode exhibited a high reversible capacity of 294 mAh/g at 0.05 A/g, which was twice that of the oxygen-deficient carbon (BHC-CO_2_-H_2_) anode. These results indicate the importance of surface functional groups in this application. The BHC-CO_2_ anode also showed excellent cycling stability, retaining 80 mAh/g after 2000 cycles at 1 A/g. Qualitative analyses revealed that the carboxyl groups introduced during the mechanochemical process facilitated Na^+^ adsorption on the carbon surface, significantly enhancing the overall capacity of the carbon electrode.

**Figure 10:**
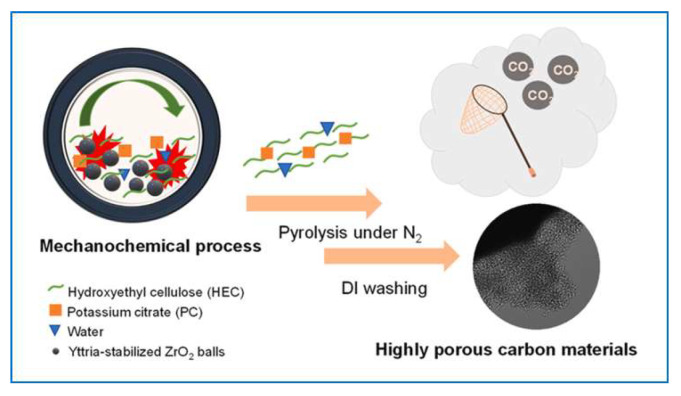
The schematic illustration of typical synthesis process of highly porous carbon materials using hydroxyethylcellulose as a carbon source [124]. Reproduced with permission from Ref. [124]. Copyright © 2023, Elsevier Inc.

Huang et al. [123] used ball milling to incorporate oxygen-containing functional groups to the surface of cellulose nanofibers. They successively dispersed native cellulose by milling it with succinic anhydride in DMSO. The resulting succinylated nanofibers could be afterward dispersed in water by solvent exchange. Elsewhere, in [118], using the mechanochemical ball milling of carbonized microcrystalline cellulose with dry ice, it was possible to obtain hard carbon with an exceptionally high oxygen content (19.33 at.%) and a predominant amount of carboxyl groups on the carbon surface. Jaroniec et al. [124] described a mechanochemical process for the synthesis of highly porous carbons through a simple and environmentally friendly treatment of a cellulose-based precursor, followed by carbonization and mild activation processes (Figure 10). Potassium citrate was used as a mild chemical activator. The obtained chemically activated carbon exhibited a large ultramicropore volume (0.37 cm^3^/g) and a high CO_2_ adsorption capacity of 4.2 mmol/g at 25 °C under 1 bar. Their article highlights the possibility of mechanochemical treatment of cellulose-based materials as a potential method for easy production of sustainable carbons with enhanced porosity, particularly an increased volume of the smallest micropores (ultramicropores). The production of highly microporous carbons from selected precursors, such as cellulose- and tannin-based precursors, may involve a simple and environmentally friendly mechanochemical process followed by physical or chemical activation.

### 5.3. Biomass Waste-Derived Carbons

**Figure 11:**
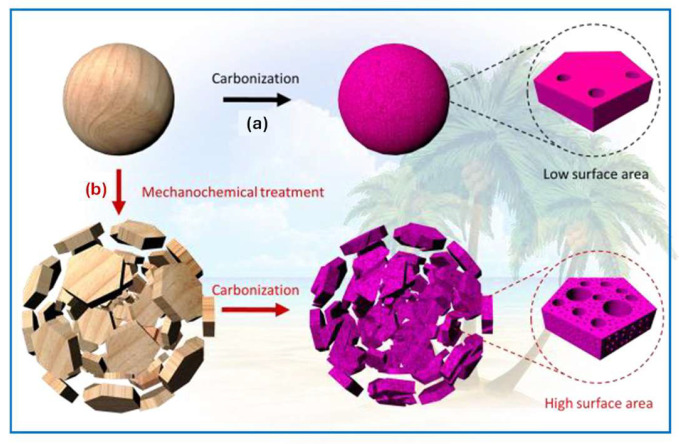
(**a**) Preparation of conventional carbon materials with low surface areas by carbonization of agricultural wastes. Physical or chemical activation is required to increase their porosity. (**b**) Preparation of high-surface-area carbon materials (HSACs) via mechanochemical treatment and subsequent carbonization. Upon the ball milling process, the monolithic agricultural wastes are smashed into tiny microparticles with a higher surface area and abundant bulk defects. This approach leads to highly porous carbons after subsequent carbonization [125]. Reproduced with permission from Ref. [125]. Copyright © 2017, American Chemical Society.

In [125], an interesting mechanochemical strategy for producing highly porous carbons (HSACs) is reported. HSACs were obtained from coconut shells, walnut shells, and bamboo waste via mechanochemical grinding followed by carbonization at 800–1000 °C under nitrogen for 3–18 h, and they exhibited SSAs up to 1771 m^2^/g and pore volumes up to 1.88 cm^3^/g. The core of the process was high-energy ball milling, which created numerous surface and internal defects in the material. The overall synthesis process is shown in Figure 11. This strategy offers several significant advantages, including cost-effectiveness and sustainability. Importantly, low-value agricultural by-products, such as coconut shells, nutshells, and bamboo waste, can be successfully converted into porous carbons. Moreover, the prepared carbon products are in the form of micro/nanoscale powder, which is advantageous for applications such as electrode materials for supercapacitors and lithium–sulfur batteries. The micro/nanoscale structure ensures better binding on current collectors and easier electrolyte access during charge–discharge cycles.

Elsewhere, hierarchical porous carbons (HPCs) were synthesized from clean, dried *Magnolia denudate* flowers through a solvent-free ball-milling method combined with potassium bicarbonate activation [126]. By varying the mass ratio of KHCO_3_ to biochar, the resulting HPCs featured high SSAs ranging from about 1640 m^2^/g to about 2150 m^2^/g and hierarchical pore structures, including micro-, meso-, and macropores. This method holds promise for the eco-friendly conversion of other biomass waste into valuable carbon materials.

Adeniran and Mokaya [7] described a method for obtaining biochar from wood sawdust using mechanical compaction prior to thermochemical activation. Very high values of pore structure parameters were obtained for these carbons, i.e., SSA close to 4000 m^2^/g and total pore volume close to 2.5 cm^3^/g. Elsewhere, ball milling was shown as an effective method to reduce the particle size of sago pith waste-derived activated carbon treated with KOH and KMnO_4_ as activating agents [127]. For instance, the high-energy ball milling of KOH-activated carbon at 500 rpm for 150 min enhanced its SSA from 170 to 497 m^2^/g. Highly porous carbons with an SSA up to 1293 m^2^/g and pore volume up to 1.4 cm^3^/g were obtained from waste tobacco straw using nano-ZnO as both a template and activator via ball milling, followed by a carbonization–activation step at 800 °C [128]. In brief, after cleaning the tobacco straws, they were crushed into powdered form and next mixed with nano-ZnO in a weight ratio of 1:1 by ball milling at 350 rpm for 2 h. During the subsequent high-temperature thermal treatment, the added ZnO served as an activator, reacting with carbon atoms (ZnO + C = Zn + CO), and simultaneously as a hard template (site-occupying effect), leading to well-developed micro–mesoporous carbon structures. Interestingly, N-doped porous carbons were synthesized from a traditional Chinese food called yuba, which is the soybean protein–lipid film of heated soybean milk [129]. Yuba contains more protein (wt. 53%) than that of most high-protein biomass, such as soybean or egg white. The authors investigated the effect of pre-carbonization ball-milling of yuba powder and the post-synthesis ball milling effect on the resulting carbon and its oxygen reduction reaction (ORR) performance. They observed that carbonization–activation of yuba powder and a ZnCl_2_ mixture at 850 °C led to porous carbon (SSA of 832 m^2^/g) with a high nitrogen content (4.29%) and superior ORR activity, comparable with that of a commercial 20% Pt/C catalyst. It was demonstrated that pre-ball-milling (up to 18 h) of the precursor is beneficial for the electrochemical application of the resulting carbon because of its increased surface area and large number of active sites. Therefore, the limiting diffusion current density of the sample was improved. In contrast, the post-milling process produced small particles of carbon, which tended to aggregate; thus, the obtained samples showed a reduction in the surface area and the number of active sites. Elsewhere, ball milling was used in the two-step process of an easy conversion of protein-rich enoki mushroom biomass into nitrogen-doped porous carbon nanomaterials containing carbon nanotubes (CNTs) [130]. The precursor with the addition of CNTs (mass ratio of 2:1) was milled at 400 rpm for 5 h and subjected to carbonization at 900 °C. The ball milling of the fungi led to small carbon particles of 2.8 μm with irregular morphology. Moreover, the addition of CNTs restricted the agglomeration of the particles. The resulting porous carbon material exhibited an SSA of 305 m^2^/g and showed excellent performance as a metal-free ORR catalyst. The ORR half-wave potential measured on the final composite material was around 0.81 V in alkaline medium, only slightly lower than that on the 20 wt% Pt/C catalyst (0.86 V).

Nanobiochar is a nanostructured biochar that might exhibit superior properties and find applications in diverse modern technologies. It can be obtained from bulk biochar using techniques such as ball milling, centrifugation, sonication, and hydrothermal synthesis [16]. Moreover, “technological nanobiochars”, or biochar nanocomposites with improved functionalities, can be obtained by various modifications of nanobiochars. Compared to bulk biochars, nanobiochars offer significantly higher surface areas (0.4–97 times), pore sizes (0.1–5.3 times), total pore volumes (0.5–48.5 times), and surface functionalities, leading to, for example, enhanced adsorption properties for pollutant removal from air or water [131,132]. Additionally, nanobiochars exhibit promising catalytic properties and may be used in sensors, fillers, drug delivery systems, enzyme immobilization, and polymer production. A comprehensive review [16] discusses the advantages and limitations of nanobiochars compared to bulk biochars, detailing their synthesis processes, pollutant adsorption mechanisms, and future directions. It was demonstrated that mechanochemistry is particularly effective for producing nanobiochars; they can be obtained from various feedstocks (i.e., rice husk, bagasse, wheat straw, okra stem, wood, etc.). For instance, Naghdi et al. [131] obtained nanobiochar from pine wood-derived biochar by a ball milling-assisted method with carbonization. They studied how different ball milling parameters, such as rotational speed, milling time, ball type, and ball-to-powder mass ratio, affected the inherent characteristics of the final nanobiochars. After optimizing the parameters (time of 1.6 h, speed of 575 rpm, and 4.5 g/g ball-to-powder ratio) and other factors, porous nanobiochar with an average particle size of 60 nm and an enlarged SSA (47 m^2^/g) in comparison to biochar (3 m^2^/g) was obtained via an energy-efficient process that involved a low temperature and low energy consumption. It was demonstrated that the prepared nanobiochar had a higher adsorption capacity for the removal of carbamazepine from water compared to raw biochar.

## 6. Applications of Biomass-Derived Carbons

Over the more than two decades of the 21st century, porous carbons derived from biomass have gained significant importance in various applications related to energy production and storage, as well as environmental protection [116]. They are used in gas adsorption/separation, catalysis, and electrochemical energy storage, and are attractive electrode materials for supercapacitors and various hybrid devices [2,9,133]. Table 2 presents examples of biomass-derived raw materials, the resulting carbon materials, their specific surface areas (SSAs), and their proposed practical application [133,134,135,136,137].

### 6.1. Gas Adsorption on Mechanochemically Synthesized Carbons

**Figure 12:**
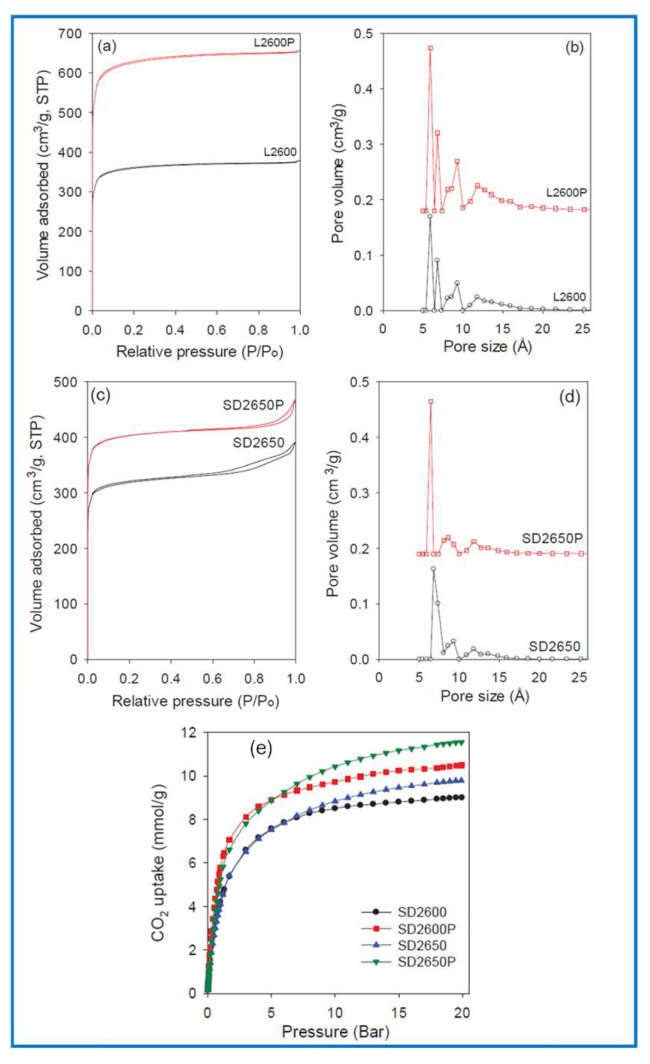
(**a**) Nitrogen adsorption isotherms and (**b**) pore size distribution (PSD) curves of sawdust-derived mechanochemically activated (SD2 x P) and conventionally activated (SD2 x) carbons prepared at x = 600 or 650 °C and a KOH/carbon ratio of 2. (**c**) Nitrogen adsorption isotherms and (**d**) corresponding pore size distribution curves of lignin-derived conventionally activated (L2600) and mechanochemically activated (L2600P) carbons prepared at 600 °C using a KOH/carbon ratio of 2. The PSD curve of the L2600P is offset (y-axis) by 0.18 cm^3^/g. (**e**) CO_2_ adsorption isotherms at 25 °C and pressure range 0–20 bar for sawdust-derived mechanochemically activated (SD2 x P) and conventionally activated (SD2 x) carbons prepared at x = 600 or 650 °C and a KOH/carbon ratio of 2 [134]. Reproduced with permission from Ref. [134]. Copyright © 2015, WILEY-VCH Verlag GmbH & Co. KGaA, Weinheim.

The porosity of adsorbents plays a crucial role in CO_2_ adsorption, especially the presence of micropores ranging from 0.34 to 0.70 nm, which significantly contribute to the CO_2_ adsorption capacity of porous carbon materials under ambient conditions (1 bar, room temperature) [151]. Various methods (e.g., templating, activation, mild pyrolysis) have been developed to synthesize porous carbon adsorbents with a high volume of micropores. For instance, a mechanochemical method used to produce porous carbon from tannic acid and iron chloride (FeCl_3_·6H_2_O) through carbonization, iron extraction, and KOH activation led to efficient CO_2_ adsorbents [95]. This carbon showed high CO_2_ adsorption of 5.8 mmol/g at 0 °C and 3.4 mmol/g at 25 °C under 1 bar pressure. Moreover, the initial isosteric heat of adsorption and selectivity for CO_2_/N_2_ were as high as 63.16 kJ/mol and 22.7 (0 °C), respectively. The adsorbent could be reused, indicating great potential for practical applications. Elsewhere, in [134], mechanochemical activation generated biomass-derived carbons with a very high CO_2_ storage capacity due to their highly developed microporosity compared to analogous conventional activated carbons with similar pore sizes. This mechanochemical activation, also known as the densification process, involves compressing a mixture of an activating agent (e.g., KOH) and biomass hydrochar into pellets/disks under 740 MPa pressure before thermal activation. Despite the increase in SSA and pore volume by 25% to 75% compared to conventional activated carbons, nearly all porosity in mechanochemically activated biomass-derived carbons (from sawdust and lignin) comes from ultramicropores (0.58–0.65 nm), resulting in a great increase in their CO_2_ adsorption capacity at 25 °C and low pressure (≤1 bar). Apparently, sawdust-derived carbon afforded a record CO_2_ adsorption at 25 °C of 5.8 mmol/g under 1 bar and 11.6 mmol/g under 20 bar (Figure 12). They are thus good candidates for pressure swing adsorption and vacuum swing adsorption processes. Their excellent volumetric CO_2_ adsorption properties result from high packing density and high contribution of the smallest pores in their structure.

Mesoporous carbons, including OMCs, exhibit CO_2_ adsorption capacity of about 1.5–2.5 mmol/g at 20/25 °C (3.0–3.5 mmol/g at 0 °C) under 1 bar due to the relatively low contribution of micropores in their structures [92,152,153]. Higher values from the range show mesoporous carbons doped with nitrogen. Nitrogen can be quite easily incorporated into the carbon structure in the stage of mechanochemical synthesis via addition of a nitrogen source to the milling system or by a post-synthesis treatment (e.g., with ammonia) [92,152]. Comparable CO_2_ adsorption properties showed porous carbons derived from chestnut tannins activated with potassium citrate and zinc chloride [154]. A higher adsorption capacity of about 4.7 mmol g^−1^ of CO_2_ at 0 °C under 1 bar was observed for mechanically synthesized carbons from tannic acid using potassium oxalate as an activating agent [94]. A combination of activation using potassium oxalate and salt templating may lead to enhanced porosity of carbons, providing a high H_2_ adsorption capacity (e.g., 13.2 mmol g^−1^ at −196 °C under 1 bar) [94].

### 6.2. Catalysis

**Figure 13:**
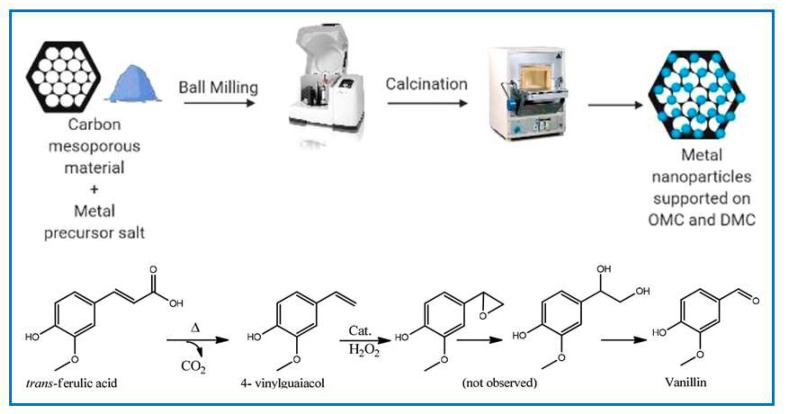
Mechanochemical functionalization of the mesoporous carbons and conversion of trans-ferulic acid into vanillin via formation of 4-vinylguaiacol as an intermediate [158]. Reproduced with permission from Ref. [158]. Copyright © 2021, American Chemical Society.

Many studies highlight the suitability of biomass-derived carbons for catalytic applications [155]. These carbons can serve as catalysts or supports for other catalysts, e.g., metal nanoparticles. The catalytic efficiency of heterogeneous systems is strongly influenced by the raw materials used in their synthesis [156]. An ideal catalyst support should provide strong metal–support interactions, resist sintering and aggregation of catalyst nanoparticles, minimize catalyst loss during reactions, and assure efficient mass transport. In this regard, ordered mesoporous carbons (OMCs) and their composites have gained attention due to their high surface area, tunable pore sizes, large pore volumes, and abundant oxygen-containing functional groups that facilitate the dispersion of metal catalysts. Their ordered mesopores enhance the transport of large molecules to active sites, like narrow pores. As a result, OMCs have been widely explored as supports in acid-catalyzed reactions, photocatalysis, and other catalytic processes [90,136,157,158]. For instance, OMCs with highly dispersed Ni particles (Ni-OMCs) synthesized via a ball milling-assisted method showed high efficiency for the selective hydrogenation of bulk molecules (~2 nm) [136], achieving a 98% yield for cyclohexane and 96% for 1-octadecane, significantly outperforming Ni-decorated activated carbon (97% and 65%, respectively). This improved performance is attributed to the synergistic effect arising from the OMC structure (large, uniform pores and high surface area) and the catalytic properties of well-dispersed Ni nanoparticles. Additionally, these catalysts can be magnetically recovered and reused at least five times without significant loss of activity. In a study conducted by Wang et al. [90], their synthesized Ru-OMC exhibited good catalytic activity in the selective oxidation of benzyl alcohol to benzaldehyde (dehydrogenation process). The oxidation process performed without the catalysts or in the presence of bare OMC did not lead to the desired product. In contrast, using Ru-OMC, they observed moderate conversions (11.3–13.6%) and good selectivity towards benzaldehyde. Cu- or Fe-decorated mesoporous carbons derived from mimosa tannins were used as heterogeneous catalysts for the conversion of trans-ferulic acid into vanillin [158]. OMCs and disordered mesoporous carbons (DMCs) containing 0.5 wt.% iron or copper were prepared using a simple and fast mechanochemical synthesis approach (Figure 13). The highest selectivity towards vanillin (47–50%) stability upon repeated measurements was achieved for Cu-decorated OMC calcined at 500 °C.

**Figure 14:**
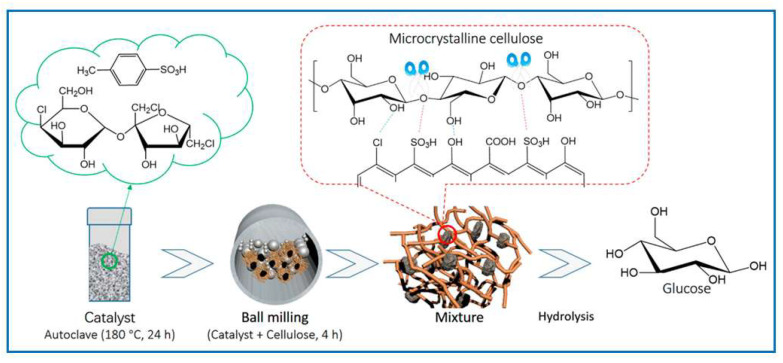
Scheme of synthesis of the carbonaceous solid acid catalyst and cellulose hydrolysis in aqueous solutions [159]. Reproduced with permission from [159]. Copyright © 2018, American Chemical Society.

In the work reported by Qui et al. [159], an efficient solid carbon acid catalyst containing –Cl and –SO_3_H groups was synthesized by solvent-free carbonization of sucralose and p-toluenesulfonic acid, followed by mechanical mixing with cellulose for 4 h. Subsequently, a rapid hydrolysis of cellulose in water was performed, as shown in Figure 14. This simple and effective method enabled high glucose yields in a short time. Additionally, the influence of the catalyst used and pretreatment via ball milling on the hydrolysis of cellulose to glucose was investigated. It was demonstrated that the catalyst exhibited satisfactory catalytic activity, leading to a high yield of glucose obtained from the hydrolysis of cellulose in aqueous reaction systems.

In mechanochemical synthesis, the composition of the biomass used, particularly its ash content, inorganic constituents, moisture, and heteroatoms, plays an important role in determining the structure and properties of the final materials, including catalysts. Biomass ash, composed of various inorganic oxides, can either catalyze or interfere with target reactions, while moisture affects milling efficiency and the formation of specific phases. Ash composition varies significantly depending on the biomass source, which in turn can lead to major differences in material performance. For instance, oxides such as silica (SiO_2_) and alumina (Al_2_O_3_) can influence the development of specific crystal phases. In some synthetic mechanisms, ash content can be advantageous, i.e., it can promote porosity in activated carbon, while in others, high ash levels may reduce surface area or introduce impurities that lower the material’s quality. Heteroatoms present in the biomass can also be incorporated into the product’s structure or modify its functional characteristics. Overall, ash and other heteroatom-based components can influence reactivity and porosity, and may introduce unwanted impurities into the catalyst structure [6,28].

### 6.3. Electrochemical and Energy-Related Applications

Carbons derived from biomass hold great potential for applications in electrochemical energy storage and conversion systems, e.g., supercapacitors and fuel cells, owing to their hierarchical pore structure, high specific surface area, and strong ability to support active materials. For instance, the kinetics of proton exchange membrane fuel cells (PEMFCs) are often limited by the oxygen reduction reaction (ORR) at the cathode, which can be improved using highly efficient electrocatalysts supported on porous carbons with hierarchical structures. Similarly, improving the performance of supercapacitors requires hierarchical porous carbon electrodes with high surface areas capable of storing large amounts of charge. Hierarchical porous carbons (HPCs) synthesized from fallen flowers via solvent-free ball milling combined with potassium bicarbonate activation showed high specific capacitances of 302.7 F/g at a current density of 0.5 A/g in 6 M of a KOH electrolyte [126]. A symmetric supercapacitor comprising these HPC electrodes demonstrated excellent stability, retaining 100% capacitance after 5,000 charge/discharge cycles and 90.5% after 12,000 cycles. Additionally, nitrogen-doped HPCs showed superior electrocatalytic activity and methanol tolerance in ORR compared to commercial Pt/C catalysts. Apparently, this method of producing HPCs could be extended for the eco-friendly processing of various other types of biomass waste into useful carbon materials.

Willow catkins are an easy and accessible source to produce hierarchically porous carbon microtubes after carbonization and activation with KOH [160]. The resulting materials retained the natural tubular morphology of willow catkins and exhibited a well-developed hierarchical porous structure, with a significant amount of nitrogen preserved from the biomass. When tested as an electrode in a three-electrode system using 6 M aqueous KOH, the carbon material demonstrated a high gravimetric capacitance of 292 F/g at a current density of 1 A/g, along with a strong rate capability, maintaining 83.5% capacitance at 10 A/g. Additionally, nitrogen-doped nanoporous carbons were synthesized via a solvent-free, mechanically induced synthesis from lignin, urea, and potassium carbonate, and they showed excellent performance for electrochemical energy storage as supercapacitor electrodes [116]. The carbon-based electrodes exhibited high specific capacitances in 1 M aqueous Li_2_SO_4_ electrolyte (177 F/g) and ionic liquid (192 F/g). Elsewhere, in [161], porous nitrogen-doped carbon nanoparticles were prepared from mechanically ground pine nut shells, a source of abundant biomass waste. These were subsequently activated with KOH and melamine. The activation process resulted in the formation of porous nitrogen-doped carbon nanosheets. In a two-electrode configuration, supercapacitors with electrode materials prepared from the synthesized carbon nanosheets exhibited an excellent specific capacitance of 324 F/g at 0.05 A/g, exceptional performance of 258 F/g at 20 A/g, and remarkable cycle stability of 94.6% after 10,000 cycles at 2 A/g in a 6 M aqueous KOH electrolyte. Zhang et al. [162] produced a low-cost SiO_2_-x/C composite from needle coke via a simple mechanochemical method. This process minimized energy utilization by harnessing the heat generated during ball milling to fuse nanoparticles into a 3D porous structure. The SiO_2_-x/C composite was employed as an anode material for lithium-ion batteries. Uniform carbon distribution within the composite provides good electrical conductivity, significantly enhancing its cycling stability. The anode delivered a high reversible capacity of about 550 mAh/g after 180 cycles. Even at a current density of 800 mA/g, it maintained a discharge capacity of 390 mAh/g. Considering the low cost of raw materials and the simplicity of the synthesis method, the composite presents a promising candidate for lithium-ion battery anodes. Table 3 presents porous carbons derived from biomass obtained using various activation methods for applications in supercapacitors, along with their SSA, pore volume, and maximum specific capacitance.

## 7. Conclusions and Perspectives

Renewable biomass can serve as an alternative to limited fossil fuel resources for producing chemicals and porous materials. However, challenges persist regarding the efficiency and environmental sustainability of biomass conversion. Green chemistry, also known as sustainable chemistry, focuses on designing processes and products that minimize the generation of harmful substances. Mechanochemistry aligns closely with green chemistry principles as it simplifies technological procedures, eliminates laborious processes involving gases and reagents, and may serve as a key technology in waste biomass processing [195]. Thus, mechanochemistry appears to be an eco-friendly and cost-effective method for producing carbon materials from various biomass sources. Diverse cost-effective methods for the synthesis and modification of biochars, such as metal impregnation and physical activation, have been recently proposed to enhance their specific properties and applicational performance. This review presents recent advances in the synthesis of porous carbon materials through the ball milling of biomass and biomass wastes. It covers the physicochemical properties of the resulting carbons, with a particular focus on their adsorption, catalytic, and electrochemical properties. The mechanochemical synthesis of porous carbons offers several advantages over conventional solution-based methods. This approach enables faster biomass conversion while eliminating steps such as solid–liquid separation and drying. The abundance of renewable biomass makes it a sustainable carbon source, suitable to produce adsorbents and supports for metallic or metal-free catalysts. Moreover, this technique allows for the easy functionalization of the resulting materials, including incorporation of heteroatoms, functional groups, metal nanoparticles, and surface defects that enhance the reactivity of the milled sample [196].

Future market demand emphasizes highly efficient, selective, and cost-effective adsorbents and catalysts produced using facile, economical, and ecological methods. Mechanochemistry offers an environmentally friendly approach for developing and producing functional nanoporous materials. Given their wide range of applications, this method is particularly well suited for synthesizing such materials using inexpensive and sustainable resources [30]. The significant reduction in solvent consumption associated with ball milling contributes to its lower costs and reduced environmental impacts [197]. However, post-synthesis purification is usually required, similar to solution-based methods. Moreover, mechanochemical devices such as ball mills and extruders face several challenges that limit their scalability and industrial applicability. These include issues with temperature control, contamination, and energy efficiency, all of which affect the economic viability of large-scale mechanochemical processes. The quantities of mechanochemically synthesized functional materials currently range from a few grams to hundreds of kilograms. Mechanochemical synthesis conditions can be easily adjusted, enabling the production of materials on a larger scale. However, as synthesis scales increase, challenges such as uneven mass and heat transfer effects can degrade the structure and properties of the final products. Moreover, heat generated during milling can cause unwanted side reactions or phase changes, which limit options for precise control. Careful optimization of screw design and temperature profiles in extruder setups are more complex than in ball mills. Despite the potential for scale-up, further production increases may also be hindered by technical difficulties associated with the construction of safe-operating high-speed mills or extruders with a high production capacity; the throughput may be especially limited for low-density materials like plant-based feedstocks. Moreover, the high energy consumption of mechanochemical processes presents a barrier to large-scale processing. Combining mechanical milling with other technologies could help reduce energy consumption. However, one should note that high-energy milling generates significant vibrations, requiring robust infrastructure and potentially complicated integration into existing processes. Another important issue is contamination; the wear of milling accessories can introduce impurities, especially when reactive materials are involved, posing challenges for high-purity applications.

This article provides a critical review of the current state of knowledge on milling technologies for biomass processing. For instance, mechanical milling can be applied for the pretreatment of lignocellulosic biomass, achieving yields comparable to traditional acid or base pretreatment methods while maintaining the simplicity of solvent-free mechanochemical processes. Ball milling pretreatment enhances biomass conversion efficiency by influencing crystallinity, polymerization degree, surface area, thermal stability, and particle size. Mechanochemistry also opens new avenues for the design and synthesis of biomass-derived materials, particularly ordered mesoporous carbons, hierarchical porous carbons, and metal–carbon composites. This method has enabled the production of diverse, highly porous carbons from tannins, cellulose-based biomass, dried flowers, coconut shells, nutshells, bamboo waste, sawdust, and other natural sources. Mechanochemical synthesis usually involves preliminary precursor preparation and processing, including reactions with other additives, mixing with activating agents, conversion, incorporation of functional additives, etc., prior to undergoing thermal treatment (carbonization). The mechanochemical processing of biomass facilitates intimate mixing and enhances reactivity, often under solvent-free conditions. However, in most cases, the milled mixture must undergo thermal treatment to achieve the desired structural and functional properties in the final material. Fundamental research on mechanochemical synthesis of nanoporous carbon materials is crucial for further advancement of nanomaterials. Many questions remain, such as how biomass structure changes under mechanochemical treatment. We hope that soon, novel carbon-based adsorbents, carbon-supported catalysts, and other nanocomposite materials derived from inexpensive biomass wastes, as well as practical large-scale mechanochemical technologies, will emerge.

## Figures and Tables

**Table 1:** Calculated adsorption parameters for various tannin-based materials obtained with or without metal salt, using different triblock copolymers as templates, and under various carbonization temperatures [84].

Sample	V_SP_ (cm^3^g^1^) *	S_BET_ (m^2^g^1^) ^†^	V_mi_ (cm^3^g^1^) ^ǂ^	S_mi_ (m^2^g^1^) ^§^	w_KJS_ (nm) ^#^
C@Tannin-Zn	0.23	514	0.20	469	–
C@Tannin-F127	0.36	395	0.11	245	–
OMC@F127_0.4_-800	0.59	773	0.19	475	7.3
OMC@F127_0.6_-800	0.76	1057	0.24	601	7.8
OMC@F127_0.8_-800	0.58	621	0.12	293	6.9
OMC@F127_1.0_-450	0.66	547	0.08	180	8.6
OMC@F127_1.0_-600	0.67	869	0.17	412	8.2
OMC@F127_1.0_-800	0.69	734	0.16	390	7.8
Ni-OMC@F127_0.8_-450	0.96	996	0.19	464	6.9
Ni-OMC@F127_0.8_-600	0.73	769	0.15	356	7.8
Ni-OMC@F127_0.8_-800	0.52	558	0.14	355	9.2
OMC@F38_0.8_-800	0.49	722	0.16	381	5.3
OMC@F68_0.8_-800	0.58	770	0.15	350	5.3
OMC@F87_0.8_-800	0.62	765	0.17	412	5.9
OMC@F88_0.8_-800	0.61	733	0.13	316	5.7
OMC@P65_0.8_-800	0.60	851	0.15	340	4.2
OMC@P85_0.8_-800	0.66	770	0.17	405	6.6
OMC@P103_0.8_-800	0.76	825	0.19	466	7.5
OMC@P123_0.8_-800	0.73	811	0.13	310	5.4
OMC@Bj78_1_-800	0.89	695	0.16	382	17
OMC@TritonX100_0.8_-800	0.50	782	0.17	407	5.0
OMC@(F127 + Ph_3_P)_0.8_-800	0.57	496	0.10	244	10.4

Notation: * Single point pore volume at relative pressure of 0.98. ^†^ Specific surface area calculated using the BET equation in the relative pressure range of 0.02–0.05. ^ǂ^ Micropore volume. ^§^ Micropore surface area calculated using the carbon black STSA t-plot equation within the thickness range of 0.354–0.500 nm. ^#^ Pore width from the distribution maxima calculated according to the Kruk-Jaroniec-Sayari (KJS) method using carbon black as reference.

**Table 2:** Comparison of SSA and application area of porous carbons from different biomass precursors synthesized using different methods.

No.	Raw Material	Type of Carbon Material	SSA, m^2^ g^−1^	Application	Ref.
1.	Abundant radish	N-O-P co-doped carbon aerogel	1649	For high-performance supercapacitors	[138]
2.	Cabbage leaf waste	Carbon aerogels	536	For supercapacitors and oil/water separation	[139]
3.	Catkins	Hollow carbon fiber sponge	438	Absorbent for oils and organic solvents	[140]
4.	Cellulose	A N-doped carbon aerogel	1196	For high-performance supercapacitors	[141]
5.	Cellulose	Carbon aerogels	-	For adsorption of diesel oil	[142]
6.	Chitosan	N self-doped carbon aerogel	1480	For high-performance supercapacitors	[143]
7.	Cocoon	Carbon aerogels	714	As efficient catalyst for oxygen reduction reaction in alkaline medium	[144]
8.	Durian shell	Carbon aerogels	735	For removal of organic pollutants	[145]
9.	Natural cotton waste	Carbon aerogels	1160	Adsorbents for wastewater clean-up	[146]
10.	Pomelo peel	Carbon aerogels	760	Absorbent for removal of organic pollutants/oils	[147]
11.	Sugarcane	Aerogel-like carbon	390	Sensor, energy conversion and storage, and EMI shielding	[148]
12.	Wood	Carbon aerogels	1124	For pressure sensing and energy storage	[149]
13.	Waste tissue paper	Carbon aerogels	1384	Adsorbent, catalyst supports, and energy storage devices	[150]
	Mechanochemically prepared carbons				
14	Enoki mushroom	N-doped porous carbon containing nanotubes	305	For oxygen reduction reaction	[130]
15	Flowers	Hierarchical porous carbon	2148	For supercapacitors	[126]
16	Lignin	Porous carbon	2224	For CO_2_ capture	[134]
17	Lignin	N-doped porous carbon	2030	For CO_2_ capture	[135]
18	Lotus roots	Porous carbon	1400	For electrochemical capacitor and oxygen reduction reaction electrocatalysis	[136]
19	Pine wood	Nanobiochar	47	For removal of organic pollutants	[131]
20	Rice straw	N-doped porous carbon	1026	For CO_2_ capture	[137]
21	Sago pith	Porous carbon	497	For methylene blue adsorption	[127]
22	Sawdust	Porous carbon	1313	For efficient CO_2_ capture	[134]
23	Tannic acid	Porous carbon	1801	For CO_2_ capture	[95]
24	Tannic acid	Highly porous carbon	3060	For efficient H_2_ and CO_2_ adsorption	[94]
25	Tannins	Highly mesoporous carbon	1218	For efficient benzene adsorption	[96]
26	Tannins	Ordered mesoporous carbon	1000	For hydrogenation of bulk molecules	[84]
27	Tobacco straw	Porous carbon	1293	For supercapacitors	[128]
28	Yuba	Porous carbon	832	For oxygen reduction reaction	[129]

**Table 3:** Summary of various biomass-derived carbon materials synthesized using different methods for supercapacitor application as well as their specific surface area, pore volume, and specific capacitance.

Biomass Resources	Activation Method	Specific Surface Area (cm^2^ g^−1^)	Pore Volume (cm^3^ g^−1^)	Electrolyte	Highest Specific Capacitance (F g^−1^)	Ref.
Bamboo waste	HTC/KOH activation	1472	–	KOH (6 M)	301	[163]
Celtuce leaves	KOH activation	3404	1.88	KOH (2 M)	421	[164]
Cherry stone	KOH activation	1300	–	H_2_SO_4_ (2 M)	230	[165]
Coffee endocarp	Physical activation	1050	0.5	H_2_SO_4_ (1 M)	176	[166]
Cow dung	KOH activation	1984	0.91	Organic	124	[167]
D-glucosamine	HTC	598	0.34	H_2_SO_4_ (1 M)	300	[168]
Fallen flowers	KHCO_3_ activation	2148	–	KOH (6 M)	303	[126]
Fallen leaves	KOH/K_2_CO_3_ activation	2869	1.598	KOH (6 M)	242	[169]
Firwoods	Physical activation	1016	0.747	NaNO_3_ (1 M)	105	[170]
Fish scale	KOH activation	2273	2.74	KOH (7 M)	168	[171]
Fungi	HTC	80.08	0.496	KOH (6 M)	196	[172]
Fungus	HTC/KOH activation	1103	0.54	KOH (6 M)	360	[173]
Ginkgo shells	KOH activation	1775	–	KOH (6 M)	178	[174]
Hemicellulose	HTC/KOH activation	2300	∼1	H_2_SO_4_ (0.5 M)	300	[175]
Hemp	HTC/KOH activation	2287	1.45	Liquid ionic	142	[176]
Human hair	KOH activation	1306	0.9	KOH (6 M)	340	[177]
Lignin	K_2_CO_3_ activation	3041	2.13	Liquid ionic	192	[116]
Lignin	K_2_CO_3_ activation	3041	2.13	Li_2_SO_4_ (1 M)	177	[116]
Lotus roots	K_2_CO_3_ activation	1400	0.81	K_2_CO_3_	273	[136]
Microalgae	HTC/KOH activation	2190	0.94	LiCl (6 M)	200	[178]
Oil palm	Physical activation	1704	0.89	–	150	[179]
Paper pulp	HTC/KOH activation	2980	1.75	Organic	166	[180]
Peanut shell	Microwave/ZnCl_2_ activation	1634	1.39	KOH (6 M)	245	[181]
Pine nut shells	KOH activation	2093	1.05	KOH (6 M)	324	[161]
Pig bone	KOH activation	2157	0.77	KOH (7 M)	185	[182]
Pinecone	KOH activation	3950	2.395	Organic	198	[183]
Pistachio shell	Physical activation	1009	0.667	NaNO_3_ (1 M)	80	[170]
Rice husk	Microwave/ZnCl_2_ activation	1442	0.71	KOH (6 M)	243	[184]
Rubber wood sawdust	Physical activation	913	0.61	H_2_SO_4_ (1 M)	138	[185]
Sewage sludge	KOH activation	2839	2.65	Na_2_SO_4_ (1 M)	379	[186]
Silk	KOH activation	2494	2.28	Liquid ionic	242	[187]
Sunflower seed	KOH activation	2585	1.41	30 wt.% KOH	311	[188]
Tobacco straw	ZnO activation	1293	1.43	KOH (6 M)	221	[128]
Waste paper	KOH activation	416	0.225	KOH (6 M)	160	[189]
Waste tea leaves	KOH activation	2841	1.366	KOH (2 M)	330	[190]
Water bamboo	KOH activation	2352	1.11	KOH (6 M)	268	[191]
Watermelon	HTC	–	–	KOH (6 M)	333	[192]
Willow catkins	KOH activation	1586	0.78	KOH (6 M)	253	[193]
Wood saw dust	HTC/KOH activation	2967	1.35	TEABF_4_ (1 M)	236	[119]
Yeast cell	KOH activation	–	–	KOH (1 M)	330	[194]

## Data Availability

Not applicable.
